# The general stress response of *Staphylococcus aureus* promotes tolerance of antibiotics and survival in whole human blood

**DOI:** 10.1099/mic.0.000983

**Published:** 2020-10-23

**Authors:** Nisha Ranganathan, Rebecca Johnson, Andrew M. Edwards

**Affiliations:** ^1^​ MRC Centre for Molecular Bacteriology and Infection, Imperial College London, Armstrong Rd, London, SW7 2AZ, UK; ^†^​ Present address: Charing Cross Hospital, Fulham, Palace Road, W6 8RF, UK; ^‡^​ Present address: Horizon Discovery, Waterbeach, Cambridge, CB25 9TL, UK

**Keywords:** *Staphylococcus aureus*, antibiotics, tolerance, sigma factor, SigB

## Abstract

*
Staphylococcus aureus* is a frequent cause of invasive human infections such as bacteraemia and infective endocarditis. These infections frequently relapse or become chronic, suggesting that the pathogen has mechanisms to tolerate the twin threats of therapeutic antibiotics and host immunity. The general stress response of *S. aureus* is regulated by the alternative sigma factor B (σB) and provides protection from multiple stresses including oxidative, acidic and heat. σB also contributes to virulence, intracellular persistence and chronic infection. However, the protective effect of σB on bacterial survival during exposure to antibiotics or host immune defences is poorly characterized. We found that σB promotes the survival of *S. aureus* exposed to the antibiotics gentamicin, ciprofloxacin, vancomycin and daptomycin, but not oxacillin or clindamycin. We also found that σB promoted staphylococcal survival in whole human blood, most likely via its contribution to oxidative stress resistance. Therefore, we conclude that the general stress response of *S. aureus* may contribute to the development of chronic infection by conferring tolerance to both antibiotics and host immune defences.

*Staphylococcus aureus* is a major human pathogen, responsible for numerous infections that range in severity from mild to fatal [1, 2]. Many of these infections can become chronic or relapse, despite apparently appropriate antibiotic therapy [1–5]. For example, staphylococcal bacteraemia relapses in ~16 % of cases, despite high-dose intravenous antibiotics [5]. Even in the case of infections caused by drug-susceptible methicillin-susceptible *S. aureus* (MSSA), such as bacteraemia, treatment with a first choice β-lactam can fail to sterilize the bloodstream, necessitating prolonged antibiotic administration [6–8]. Treatment failure of infections caused by MSSA strains has been linked to antibiotic tolerance, a phenomenon whereby bacteria survive exposure to high concentrations of antibiotics despite not being resistant to them [6, 9–11].

In addition to surviving antibiotic exposure, the persistence of staphylococcal infections demonstrates an ability of the bacterium to survive the host immune response, which includes both innate and adaptive immunity [12, 13]. Neutrophils are particularly important in containing infection, targeting *S. aureus* for destruction via the oxidative burst, antimicrobial peptides and proteases [12–17]. However, *S. aureus* has multiple defences against oxidative stress and can attack neutrophils via leukocytic toxins, which often leads to abscess formation [17–19]. As such, *S. aureus* appears to tolerate the antibacterial effects of both therapeutic antibiotics and host defences. Understanding these tolerance mechanisms is important because it may identify targets for new therapeutic approaches that sensitize the pathogen to both antibiotics and host defences.

Like all bacteria, *S. aureus* employs numerous global regulators to enable it to adapt to its environment and tolerate stresses or cell damage [18, 20, 21]. For example, the SOS response enables *S. aureus* to repair DNA damage, whilst the CodY regulon enables the bacterium to sense and adapt to various metabolic conditions [22, 23]. The general stress response of *S. aureus* is regulated by the alternative sigma factor B (σB), which confers protection against a diverse array of stresses, including oxidants, acidic and alkaline conditions, heat, ethanol and long-chain-free fatty acids [24–28]. There is also evidence that σB regulates the production of virulence factors such as cytolytic toxins, proteases, fibronectin-binding proteins and the staphyloxanthin pigment [28–32]. In turn, this modulates staphylococcal virulence, biofilm formation and intracellular survival [30–37].

The contribution of σB to virulence and stress resistance suggests that it may enhance staphylococcal survival in the host during infection and thus promote the development of chronic and relapsing infections. However, the role of σB in staphylococcal survival during exposure to host defences is largely unclear. More is known about the contribution of σB to antibiotic susceptibility, but there are still substantial gaps in our knowledge. σB has been shown to be required for high-level resistance of MRSA strains to β-lactams and the decreased susceptibility of glycopeptide intermediate *S. aureus* (GISA) strains to glycopeptide antibiotics [6]. For example, the loss of *sigB* in MRSA or GISA strains typically leads to two–fourfold reductions in the MICs of β-lactams and glycopeptides, respectively, although larger changes have been reported [38–43]. Conversely, over-expression of *sigB* results in increased MIC values, possibly due to increased cell-wall thickness that occurs via a process requiring SpoVG [41, 42, 44] However, the role of σB in modulating antibiotic susceptibility in MSSA strains or for drugs that do not target the cell envelope, is not established. Furthermore, whilst several different stress-response mechanisms have been linked to antibiotic tolerance, a phenomenon that is independent of antibiotic resistance, the contribution of σB to the survival of bacteria exposed to antibiotics is not characterized [6, 45]. This is despite several different antibiotics triggering σB activity [46–48].

Since σB provides protection against a range of diverse stresses, we hypothesized that this sigma factor may protect drug-susceptible MSSA against the twin threats of antibiotics and host defences, the tolerance of which results in the establishment of chronic or relapsing infections.

To test this hypothesis, we decided to use the well-characterized *S. aureus* SH1000 MSSA strain, which has a functional σB regulon and the isogenic Δ*sigB* strain MJH502 [30]. SH1000 was selected because σB activity is well characterized and it is sensitive to a range of different antibiotics [30]. The σB-deficient mutant was complemented by placing the *sigB* gene into plasmid pCL55itet, where it was under the control of an anhydrotetracycline (AHT)-inducible promoter (p*sigB*) [49]. Expression was induced by the presence of 100 ng ml^−1^ AHT in the tryptic soy broth (TSB) growth medium. We chose to use an inducible promoter because native *sigB* expression is controlled by three distinct promoters within the *sigB* regulon [50]. This vector inserts stably into the *geh* locus, removing the need for antibiotic selection, which could interfere with measurements of antibiotic tolerance [49]. To control for polar effects due to the presence of the plasmid in *geh*, and any effects of AHT, we also transformed the Δ*sigB* mutant with pCL55 lacking *sigB* (pEmpty). To ensure that σB activity was restored in the complemented strain, we visually assessed levels of the staphyloxanthin pigment, since this is absent in the Δ*sigB* mutant [30, 32]. As expected, the cell pellet of wild-type *S. aureus* SH1000 had a strong yellow/orange appearance, whereas the Δ*sigB* mutant was white (Fig. 1). The presence of pEmpty had no effect on pigmentation, but p*sigB* fully restored staphyloxanthin levels (Fig. 1).

**Fig. 1.**
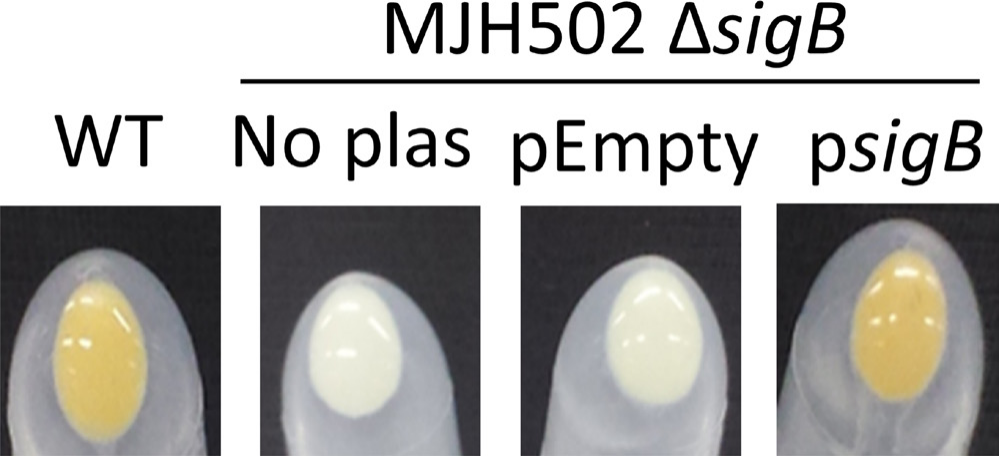
Cell pellets demonstrating pigmentation levels. *S. aureus* SH1000 wild-type (WT), MJH502 Δ*sigB* mutant without plasmids (No plas) or MJH502 transformed with pCL55itet alone (pEmpty) or pCL55itet containing the *sigB* gene under the control of the IPTG-inducible promoter, were grown in TSB and centrifuged for 3 min at 14 000 ***g*** to pellet cells. The medium was removed, and tubes inverted to enable photographs of the pellets to be taken. For the two strains with plasmids, the growth medium was supplemented with 100 ng ml^−1^ anhydrotetracycline.

We then proceeded to assess the impact of σB on antibiotic susceptibility by measuring the MIC of various classes of clinically relevant antibiotics for the wild-type, mutant and complemented strains as described previously [51–53]. For gentamicin, ciprofloxacin, vancomycin, clindamycin and oxacillin, MIC values were the same for all strains, regardless of whether they expressed σB or not (Table 1). This contrasts with what has been reported previously for MRSA and GISA strains, where a loss of *sigB* results in increased susceptibility (decreased MIC) for oxacillin and vancomycin, respectively [38–43]. It also shows that σB does not affect drug susceptibility to gentamicin, ciprofloxacin and clindamycin, which had not been demonstrated previously.

**Table 1.** MICs of various antibiotics for SH1000, Δ*sigB* mutant and complemented strains

Antibiotic	MIC (µg ml^−1^)			
	SH1000 wild-type	MJH502 (Δ*sigB*)	MJH502 pEmpty	MJH502 p*sigB*
Cloxacillin	0.25	0.25	0.25	0.25
Gentamicin	0.25	0.25	0.25	0.25
Ciprofloxacin	0.25	0.25	0.25	0.25
Vancomycin	1	1	1	1
Clindamycin	0.125	0.125	0.125	0.125
Daptomycin	2	2	2	4

For daptomycin, the MIC was the same (2 µg ml^−1^) for wild-type, MJH502 and MJH502(pEmpty) strain. This is in keeping with previous work suggesting that σB does not affect the MIC of daptomycin in MRSA or MSSA [54]. However, MJH502(p*sigB*) had a 2-fold higher MIC than the other strains (4 µg ml^−1^) (Table 1). This may be explained by slightly increased pigment expression (Fig. 1), which has been linked to decreased daptomycin susceptibility previously [55].

Next, we assessed the impact of σB on antibiotic tolerance by measuring the susceptibility of *S. aureus* to the bactericidal activity of the antibiotic panel over time [52, 53]. We exposed ~10^8^ c.f.u. ml^−1^ *S *. *aureus* from early stationary phase cultures (when σB is maximally active) to antibiotics at a concentration that was 10× the MIC of the wild-type, and measured survival over time as described previously [52, 53].

Both gentamicin and ciprofloxacin caused rapid and substantial drops in the c.f.u. counts of wild-type *S. aureus *, leading to ~100 000-fold reduction in viable bacteria after 24 h (Fig. 2a, b). However, the σB-deficient MJH502 strain was significantly more susceptible to both antibiotics, with ~1 000 000-fold fewer c.f.u. after 24 h exposure to either antibiotic when compared with the inoculum (Fig. 2a, b). Complementation of MJH502 with p*sigB* increased survival significantly, whilst pEmpty had no effect on survival (Fig. 2a, b). By contrast to gentamicin and ciprofloxacin, vancomycin did not kill wild-type *S. aureus *, with an ~twofold increase in c.f.u. counts over 24 h (Fig. 2c). However, c.f.u. counts of MJH502 did not increase, suggesting slightly increased susceptibility to the antibiotic. Complementation with p*sigB*, but not pEmpty, restored growth of MJH502, confirming the role of σB in modulating susceptibility to vancomycin (Fig. 2c). Similar to what has been reported by us and others, exposure of wild-type *S. aureus* to daptomycin resulted in an initial drop in c.f.u. counts (~100 000-fold), followed by recovery to levels similar to the inoculum (Fig. 2d) [39, 40]. However, by contrast to the wild-type strain, there was very limited recovery of the σB-deficient strain MJH502, with c.f.u. counts ~1000-fold lower after 24 h compared to the inoculum (Fig. 2d). Complementation restored the wild-type phenotype to MJH502, whilst pEmpty had no effect. Therefore, maximal recovery of *S. aureus* after daptomycin exposure appears to require σB (Fig. 2d). By contrast to gentamicin, ciprofloxacin, vancomycin and daptomycin, we did not observe any differences between wild-type and MJH502 during exposure to oxacillin or clindamycin (Fig. 2e, f). In summary, these data demonstrate that σB contributes to antibiotic tolerance in an MSSA strain, promoting the survival of *S. aureus* exposed to several, but not all, classes of clinically relevant antibiotics.

**Fig. 2.**
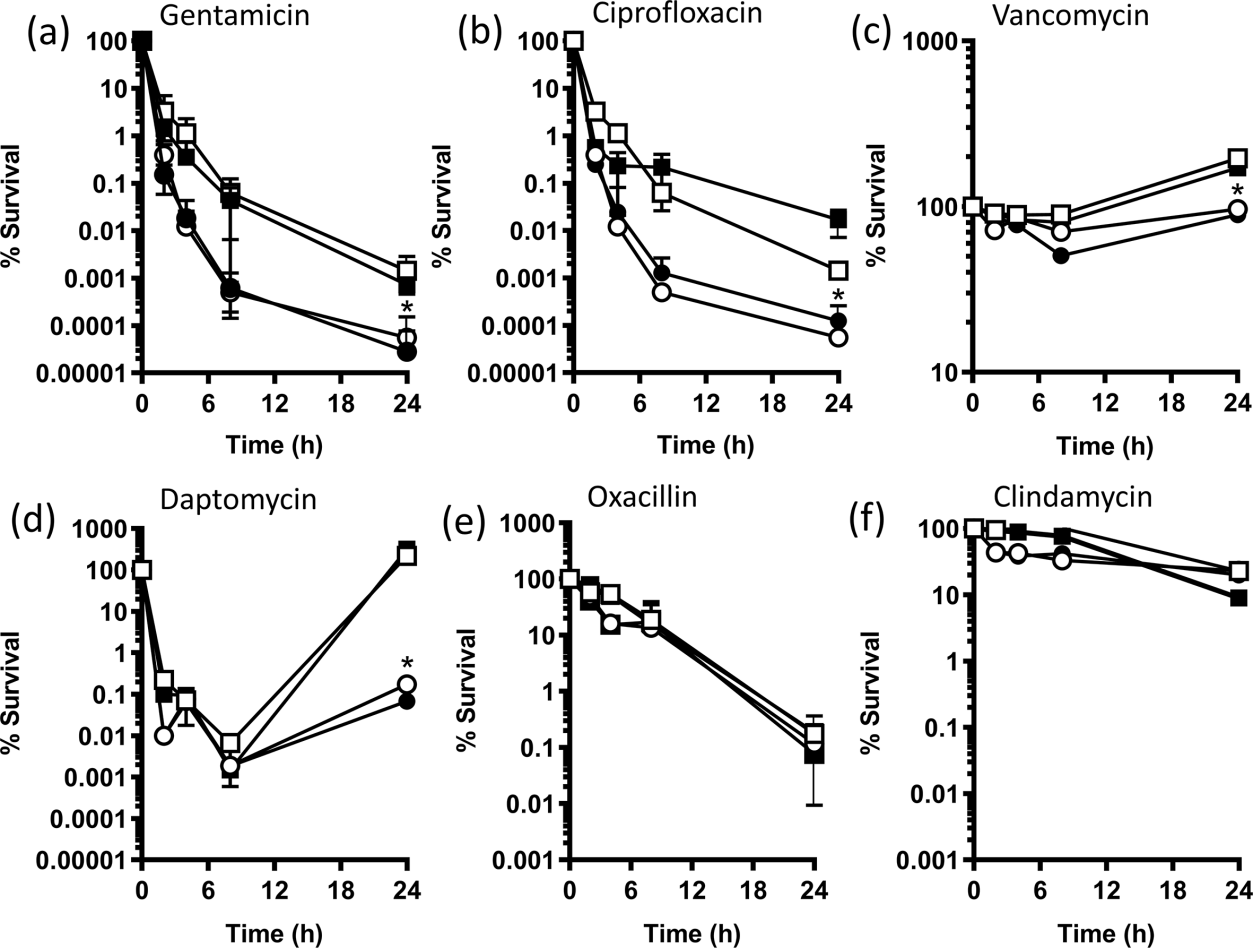
Survival of bacteria during exposure to various antibiotics. *S. aureus* SH1000 wild-type, Δ*sigB* mutant, Δ*sigB* mutant pEmpty and Δ*sigB* mutant p*sigB* were exposed to 10× the MIC of antibiotics over 24 h in Mueller–Hinton Broth and survival measured by enumeration of c.f.u. at the indicated time points. Data represent the mean of three independent experiments and were analysed by two-way repeated measures ANOVA with Tukey’s post-hoc test. Data points below (*) indicate values that were significantly different from those of the wild-type (*P*=<0.05). Error bars represent the standard deviation of the mean.

In addition to antibiotics, the other key threat to the survival of pathogens is the host immune system. Therefore, we next examined the role of σB in the survival of *S. aureus* in whole human blood, which contains a high concentration of neutrophils that are crucial in the defence against *S. aureus* [16, 17, 53, 56]. Previous work from our group and others using similar models have shown that >95 % of *S. aureus* cells are phagocytosed within 5 min by neutrophils and targeted by the oxidative burst [16, 56]. Blood was taken from healthy human volunteers as described previously [16]. Ethical approval for drawing and using human blood was obtained from the Regional Ethics Committee and Imperial NHS Trust Tissue Bank (REC Wales approval no. 12/WA/0196, ICHTB HTA license no. 12275).

c.f.u. counts of wild-type *S. aureus* in blood fell steadily during the incubation period to ~25 % of the inoculum after 6 h (Fig. 3a). Survival of MJH502 was similar to that of the wild-type for the first 4 h of incubation but was significantly lower by 6 h with just 5 % of the inoculum remaining viable (Fig. 3a). MJH502 cells containing pEmpty had a very similar survival profile to the mutant lacking the plasmid, but MjH502 p*sigB* survived at levels greater than the wild-type, confirming the role of σB in promoting staphylococcal resistance to host defences (Fig. 3a). To test whether the falls in c.f.u. counts were due to killing by the oxidative burst of neutrophils, we measured the survival of wild-type and MJH502 strain after 6 h incubation in blood treated with the NADPH oxidase inhibitor diphenyleneiodonium (DPI) chloride or DMSO alone as a solvent control [16, 17]. In keeping with previous studies, the presence of DPI increased survival of the wild-type *S. aureus* strain 2.8-fold compared to DMSO alone (Fig. 3b) [16, 17]. A larger relative increase in survival (3.7-fold) was seen for MJH502 incubated in blood containing DPI, confirming that the oxidative burst was required for most of the killing of *S. aureus* wild-type and MJH502 strains (Fig. 3b). This also suggested that the reduced survival of the SigB-deficient strain relative to the wild-type was partly due to increased susceptibility to the oxidative burst. However, since DPI did not protect the σB-deficient mutant to the same level as the wild-type, it appears that the Δ*sigB* mutant was more susceptible to non-oxidative microbicides such as antimicrobial peptides [55]. To confirm that σB promoted oxidative-stress resistance, we measured survival of our strains in H_2_O_2_. As expected from previous work, strains with functional σB survived at ~twofold higher levels than strains without σB [25, 30] (Fig. 3c). Taken together, these data demonstrate that σB promotes staphylococcal survival in whole human blood, most likely via resistance to the antibacterial activity of neutrophils.

**Fig. 3.**
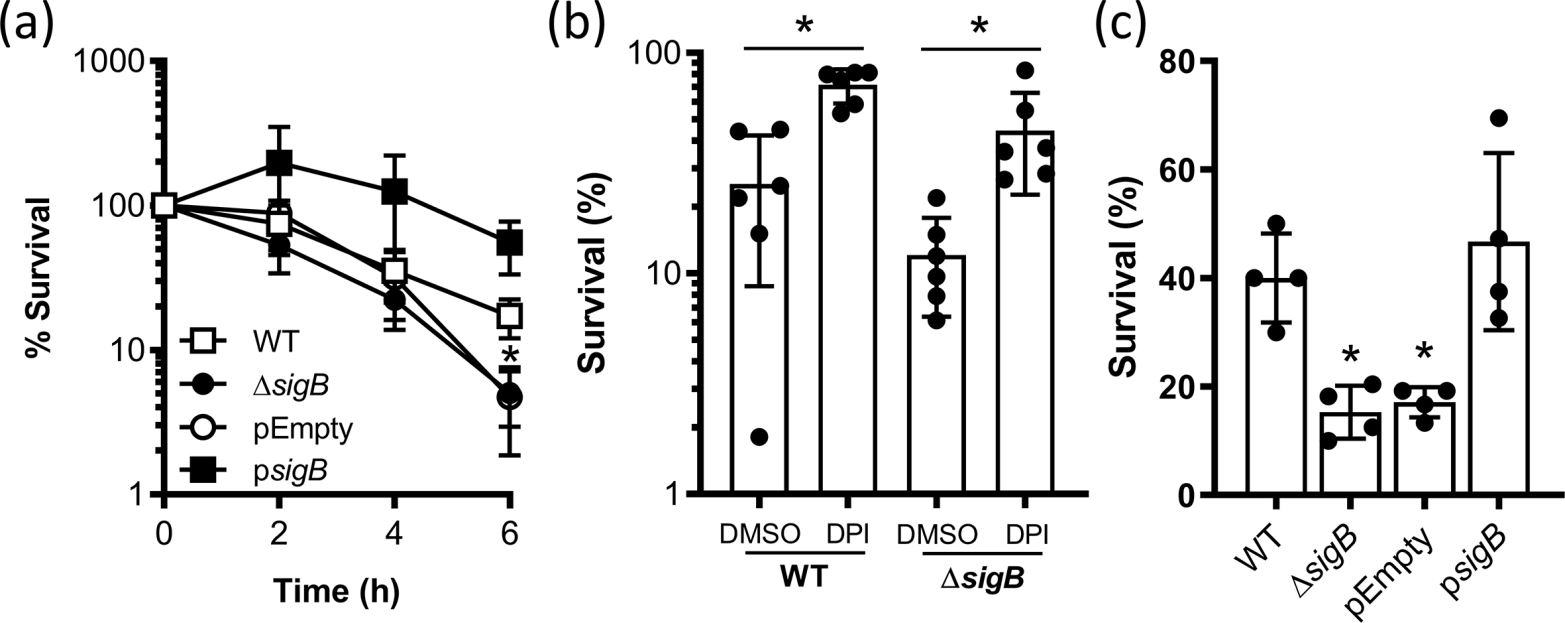
σB protects against the oxidative burst. (a) *S. aureus* SH1000 wild-type, Δ*sigB* mutant, Δ*sigB* mutant pEmpty and Δ*sigB* mutant p*sigB* were incubated in whole human blood and survival measured by enumeration of c.f.u. at the indicated time points. Data represent the mean of six independent experiments using blood from different donors and were analysed by two-way repeated measures ANOVA with Tukey’s post-hoc test. Data points below (*) indicate values that were significantly different from those of the wild-type (*P*=<0.05). (b) Survival of SH1000 wild-type (WT) or Δ*sigB* mutant (Δ*sigB*) in whole human blood containing DPI or DMSO alone (solvent control). Bars represent the mean of six independent experiments using blood from different donors. Data were analysed by two-way ANOVA with Sidak’s post-hoc test (* =<0.05). (c) Survival of *S. aureus* SH1000 wild-type (WT), Δ*sigB* mutant (Δ*sigB*), Δ*sigB* mutant pEmpty (pEmpty) and Δ*sigB* mutant p*sigB* (p*sigB*) after exposure to H_2_O_2_ for 1 h. Bars represent the mean of four independent experiments. Data were analysed by one-way ANOVA with Dunnett’s post-hoc test. *P=<0.05 relative to the wild-type. In all cases, error bars represent the standard deviation of the mean.

In summary, this study adds four classes of antibiotics and the microbicidal activity of neutrophils to the list of stresses that σB combats and contributes to our growing understanding of the roles this sigma factor plays in staphylococcal biology [57]. These findings also add to our growing appreciation of the role σB plays in pathogenesis and the development of chronic infection and provide further evidence that a single system can enhance bacterial survival during exposure to multiple different stresses [24–37].

The finding that σB enhances antibiotic tolerance, at least for some classes of antibacterial drugs indicates similarities with *Mycobacterium tuberculosis *. Pisu *et al*. found that mutants defective for σB or σE were more susceptible than wild-type bacteria to the bactericidal activity of several classes of antibiotic [58]. In keeping with this, work with *Listeria monocytogenes* found that the quinolone norfloxacin killed a Δ*sigB* mutant faster than the wild-type, suggesting that σB has a broadly conserved role in conferring antibiotic tolerance [59].

The mechanism(s) by which σB promotes tolerance of both antibiotics and the bactericidal activity of neutrophils is not yet established. It is also unclear whether the same mechanisms are employed by *S. aureus* to combat both stresses. However, σB has been shown to provide protection against oxidative stress by promoting the expression of catalase, which breaks down hydrogen peroxide and by triggering the production of the staphyloxanthin pigment [25, 30, 32]. It is unclear whether catalase promotes survival of the oxidative burst, but there is good evidence that staphyloxanthin increases the resistance of *S. aureus* to killing by neutrophils [17, 60]. For example, mutants lacking staphyloxanthin production were more susceptible to killing by purified neutrophils, as well as neutrophils in blood, unless the oxidative burst was blocked using DPI [17, 60]. In keeping with this, staphyloxanthin-defective *S. aureus* mutants are less virulent in wild-type mice, but not transgenic animals lacking the enzyme complex required for the oxidative burst [17]. In addition to ROS tolerance, staphyloxanthin provides resistance to human neutrophil defensin-1, which may explain why the σB-deficient mutant survived less well in human blood than the wild-type [55]. The important contribution of staphyloxanthin to bacterial survival in the host has led to interest in targeting the biosynthesis of the pigment as a novel therapeutic strategy that sensitises *S. aureus* to host immunity [61].

Whilst staphyloxanthin production may explain the importance of σB in resisting killing by neutrophils, the contribution of the pigment to the antibiotic tolerance of *S. aureus* is much less clear. There is evidence that staphyloxanthin promotes resistance to daptomycin and antimicrobial peptides, with over-expression of pigment leading to a twofold increase in daptomycin MIC, similar to what was observed in this study with the p*sigB* complemented strain [55]. However, the pigment has not been shown to alter susceptibility to other antibiotics. As such, it remains to be determined which members of the σB regulon mediate antibiotic tolerance.

The finding that σB activity confers tolerance of several different antibiotics, as well as the oxidative burst, suggests that inhibitors of the general stress response could be effective therapeutics. Such an approach could be used as a monotherapy to enhance immune clearance, or in combination with selected antibiotics to increase bacterial susceptibility. Small molecule inhibitors active against σB of *Bacillus subtilis* and *Listeria monocytogenes* have been reported, providing proof of concept for similar approaches to be used against *S. aureus* [62, 63].

In summary, σB promotes the survival of *S. aureus* in whole human blood and during exposure to four different classes of antibiotic, suggesting that it may provide a desirable target for novel therapeutic approaches.
